# The development of cognitive flexibility and its implications for mental health disorders

**DOI:** 10.1017/S0033291724001508

**Published:** 2024-09

**Authors:** Ke Tong, Xinchen Fu, Natalie P. Hoo, Lee Kean Mun, Chrysoula Vassiliu, Christelle Langley, Barbara J. Sahakian, Victoria Leong

**Affiliations:** 1 Cambridge-NTU Centre for Lifelong Learning and Individualised Cognition (CLIC), Nanyang Technological University, Singapore; 2 Early Mental Potential and Wellbeing Research (EMPOWER) Centre, Nanyang Technological University, Singapore; 3 Department of Psychology, University of Cambridge, Cambridge, UK; 4 Department of Psychiatry, University of Cambridge, Cambridge, UK

## Overview

**Figure 1.**
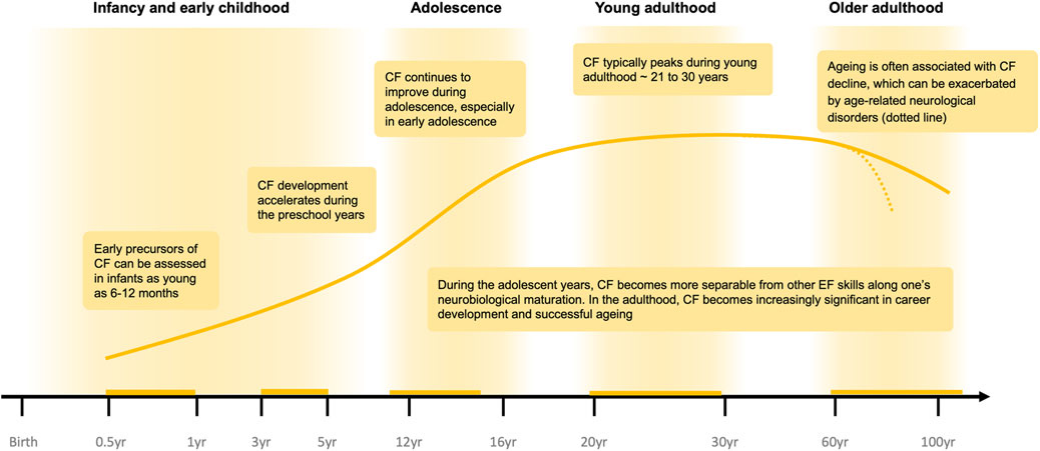
Developmental trajectory of CF maturation across the lifespan. The yellow curve is a diagrammatic representation of CF maturation across development in a healthy population (NB: this is not intended to indicate performance on a specific empirical measure). Notable milestones and inflections in the CF developmental trajectory are highlighted. These are expected to vary as a function of environmental influences and individual differences.

Cognitive flexibility (CF) represents the ability to adapt one's thinking and behavior in response to changing environmental demands (Uddin, 2021). CF is multifaceted and involves a range of skills, including attentional shifting, strategy updating, response to feedback, reversal learning, exploration, and task switching. As a core component of executive function (EF), CF works in tandem with working memory and inhibitory control to facilitate goal-oriented behavior (Friedman & Robbins, 2022). However, this editorial will focus on the development of CF and its implications for mental health disorders. CF is also impaired in a number of mental health disorders, including autism spectrum disorder (ASD) (Hughes, Russell, & Robbins, 1994), obsessive-compulsive disorder (OCD) (Gottwald et al., 2018; Vaghi et al., 2017), and schizophrenia (Murray et al., 2008). CF exhibits a prolonged maturational developmental trajectory, although early precursors of these skills can already be measured from infancy. Figure 1 provides a graphical illustration of the lifespan trajectory of CF development during infancy, adolescence, young adulthood, and older adulthood. This is also important considering that many mental health disorders begin in childhood and adolescence. Here, we discuss key environmental factors that may be important for shaping CF development across different life stages and their implications for mental health.

## Development of cognitive flexibility

### Neurobiological origins

The emergence of CF is linked to maturation of the prefrontal cortex (PFC) and inferior parietal cortex (Ezekiel, Bosma, & Morton, 2013). Although the PFC is relatively late maturing, this region already begins to undergo synaptic pruning and myelination from the first year of life (Collin & van den Heuvel, 2013). Between 1 and 2 years, there is a pronounced acceleration in the volume of prefrontal gray matter, with expansion in both cortical thickness and surface area (Gilmore et al., 2012). During the early years, external environmental stimuli are important for shaping the developmental trajectory of the PFC (Chini & Hanganu-Opatz, 2021). Specifically, factors such as caregiver interactions (Nelson, 2007), pronounced sensory deprivation (McLaughlin, Sheridan, & Lambert, 2014), prenatal exposure to substances (Mackey, Raizada, & Bunge, 2013), and early adverse experiences (Hodel, 2018) can impact PFC maturation. Neurotransmitter systems are involved in the neuromodulation of CF. For example, studies have shown that serotonin (Chamberlain et al., 2006; Skandali et al., 2018) and dopamine (Dang, Donde, Madison, O'Neil, & Jagust, 2012) both affect performance on CF tasks. In contrast noradrenaline does not seem to affect at least some CF tasks such as the CANTAB IED (Chamberlain et al., 2006).

### Measuring emerging cognitive flexibility

**Figure 2.**
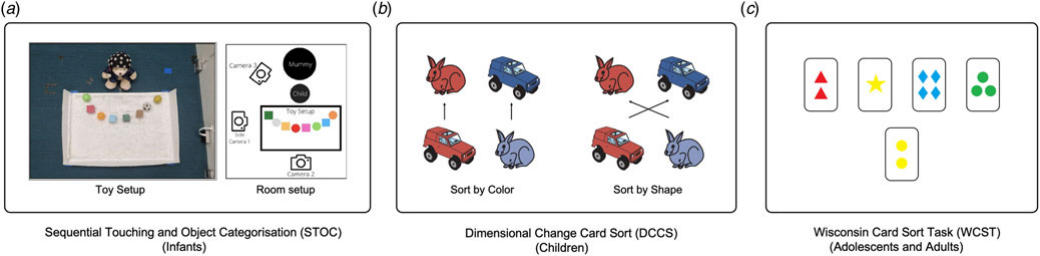
Examples of age-appropriate CF tasks for infants, children, and adults. (a) In the Sequential Touching and Object Categorization (STOC) task, infants are presented with objects that can be categorized by either a high-salience dimension (e.g. shape: balls *v.* blocks) or a low-salience dimension (e.g. material: soft *v.* hard). This task comprises three phases: phase 1- infant free play; phase 2- parent demonstration of toy material (compressibility); phase 3- infant free play. The STOC measures flexible attention set-shifting in infants' mental categorization of toy objects from 12 months of age, particularly when the shift is scaffolded by a social partner (Tan & Leong, 2023). (b) In the Dimensional Change Card Sort (DCCS) task, children sort cards based on one dimension (e.g. color) and after several trials, they are instructed to switch and sort by another dimension (e.g. shape). The task assesses their ability to shift between different sets of rules and adapt to new instructions (Zelazo, 2006). (c) In the Wisconsin Card Sort Task (WCST), participants sort a target card into one of four decks without knowing the initial sorting rule. After each sort, they receive feedback on its correctness. From this feedback, they must infer the underlying rule. After several consecutive correct responses, the sorting rule changes without notice, challenging participants to detect the shift and adjust their strategy accordingly (Tong et al., 2023).

Precursors of CF such as attention set-shifting, reversal learning, and overcoming perseveration begin to emerge during infancy. Specifically, one can assess infants' ability to overcome perseveration and engage in reversal learning from as early as 6–12 months (de Sousa, de Gil, & McIlvane, 2015). The sequential touching task (Ellis & Oakes, 2006) has been adapted as an infant CF measure to assess flexible attention set-shifting from 12 months of age, particularly when the shift is scaffolded by a social partner (Fig. 2a). Piaget's A-not-B task, a classic test of infant cognitive development, requires basic shifting, memory and inhibitory control skills, and children still make errors on this task until ~12 months of age (MacNeill, Ram, Bell, Fox, & Pérez-Edgar, 2018). Together with other core EF skills, CF development accelerates during the preschool years (Hughes, 1998) and is only thought to reach maturity during late childhood or early adolescence (Kupis & Uddin, 2023). In children, a more formal measure of rule switching is the Dimensional Change Card Sort task (DCCS, Fig. 2b), in which children are asked to sort cards according to one dimension (e.g. color, shape, or number) and this sorting role is changed after several trials. While 4-year-old children are typically able to switch successfully between different dimensions, 3-year-old children tend to perseverate on one dimension (Doebel & Zelazo, 2015). In the Intra-Extra Dimensional set shift task (IED, from Cambridge Neuropsychological Test Automated Battery), typically-developing 5-year-old children already display successful attentional set-shifting, but 7 to 18-year-old children with autism show significant CF dysfunction (Hughes et al., 1994; Langley, Sahakian, & Robbins, 2023). Childhood CF is linked to essential life outcomes such as social skills, learning, financial stability, and overall well-being (Arán Filippetti & Krumm, 2020; Broomell & Bell, 2022).

### Adolescence and young adulthood

During adolescence, performance on CF tasks continues to improve in tandem with increasing brain specialization (Kupis & Uddin, 2023). Although adolescent CF approximates adult levels from ~12 years (Huizinga, Dolan, & van der Molen, 2006), peak performance is achieved approximately between the ages of 21 and 30 (Cepeda, Kramer, & Gonzalez de Sather, 2001), during which the frontal-striatal networks maturation contributes to both cognitive and behavioral flexibility processes (Morris et al., 2016). The maturation of EF during adolescence may have interesting associations with higher levels of risk-taking behavior observed during this stage, as teenagers shift their priorities to avoid peer rejection, and inhibit health or legal concerns to engage in more risky behaviors (Blakemore, 2018). Studies employing a latent-factor approach suggest that a single unified EF factor may best describe the capacities of young children up to ~8 years of age, however by age 10, two separable EF components (putatively memory and ‘general’ EF) may be identified using statistical models (Brydges, Fox, Reid, & Anderson, 2014). In young adults, a model of three correlated factors, i.e., working memory, inhibitory control, and CF, emerges as the best EF model, termed ‘unity and diversity’ (Friedman & Miyake, 2017). Although this data fits the narrative of a developmental transition from relatively undifferentiated unidimensional EF to separable but correlated EF components, the relative paucity (and specificity) of age-appropriate tasks to measure emerging EF during infancy and childhood confounds this interpretation. Many child EF tasks require language skills to comprehend task instructions, which precludes their use in preverbal children and presents additional non-EF related task demands. There are mental health disorders which affect and impair the development of cognitive flexible thinking which start in childhood or adolescence, such as ASD (Hughes et al., 1994), OCD (Gottwald et al., 2018; Vaghi et al., 2017), and schizophrenia (Murray et al., 2008). Indeed, children and adolescents with OCD have impaired functioning at school and at home and experience severe distress. Critical cognitive domains for daily functioning and academic success are learning, memory, CF and goal-directed behavioral control. These domains, particularly learning and memory as well goal-directed control and cognitive plasticity are impaired early in the development of OCD (Gottwald et al., 2018). In adults with OCD, a severe impairment in CF has been shown. Moreover, this impairment is likely due to disruptions in the fronto-striatal circuitry (Vaghi et al., 2017) that typically subserve CF.

### Older adulthood

As individuals age, cognitive abilities typically decline, including CF (Murman, 2015). This CF decline presents as increased perseverative behaviors measurable in tests such as the Wisconsin Card Sort Task (WCST, Fig. 2c) (Ashendorf & McCaffrey, 2008). A heightened propensity for older adults to perseverate can be linked to their declined set-shifting capabilities (Ridderinkhof, Span, & van der Molen, 2002). At the neural level, the PFC experiences significantly greater gray matter volume loss during normal ageing as compared to other developmental stages (Raz et al., 1997), which contributes to the age-related decline of EF and CF. Age-related neurodegenerative diseases, such as mild cognitive impairment, dementia, and Alzheimer disease (AD), can speed up neuronal dysfunction and exacerbate cognitive declines, including CF (Guarino, Forte, Giovannoli, & Casagrande, 2020). Recent functional imaging studies investigating CF-related brain network dynamics suggest that older adults who have extended dwell time in co-activation among the lateral frontoparietal network (L-FPN, ‘executive control’ network) and medial frontoparietal network (M-FPN, ‘default’ network) show diminished CF, compared with young adults (Kupis et al., 2021).

In older adults, CF also appears to have a significant impact on motor control. Older adults demonstrate reduced motor flexibility when switching between different walking patterns, which is positively associated with higher levels of cognitive perseveration (Sombric & Torres-Oviedo, 2021). This connection suggests a shared mechanism that governs both cognitive and motor perseveration as individuals age. As older adults with diminished CF (measured by WCST) have a greater risk of losing balance and falling (Pieruccini-Faria, Lord, Toson, Kemmler, & Schoene, 2019), interventions focusing on enhancing cognitive and motor flexibility could be instrumental in maintaining the quality of life for older adults by preventing falls.

More longitudinal data are required to understand CF across the lifespan, however when examining young children, there are some data to suggest that EF assessed by a battery of tests at 24 months does relate to EF assessed at age 4 (see Miller, Galvagno, & Elgier, 2023). Stability prior to that is unclear as studies have given mixed results. There are cross sectional data on CF across the lifespan using the CANTAB IED, which shows that CF performance improves from childhood to adolescence and is optimal in early young adulthood and then remains stable until age about 50 and slowly declines during older adulthood (Langley et al., 2023).

## Potential intervention strategies to improve CF across the lifespan

### Quality of early caregiving

The quality of early caregiving is an important and modifiable factor that affects early CF development. Parental social interactive behaviors, including sensitivity, have been linked to higher CF abilities in children (Bernier, Carlson, Deschênes, & Matte-Gagné, 2012) whilst traumatic social experiences can negatively impact children's EF, including CF (Kavanaugh, Dupont-Frechette, Jerskey, & Holler, 2017). One promising parent-based EF training program is the Attachment and Biobehavioral Catch-up intervention for infants (ABC-I). This 10-session home-based program fosters nurturing and synchronous parent-child interactions (Dozier & Bernard, 2017) and has been shown to positively affect attachment security, emotion expression, and cognitive control in children whose foster parents underwent training (Bernard, Hostinar, & Dozier, 2015). These encouraging data highlight the potential for home-based programs that target parent-child relationships to enhance early EF development.

### Lifestyle factors

A range of lifestyle factors, such as sleep, exercise, nutrition, stress management, social connection, and learning new skills, can enhance EF and combat the cognitive decline associated with ageing (Beddington et al., 2008). Here, we focus on stress-management via mindfulness and exercise as an illustrative example for potential intervention. Chronic and acute stress can negatively affect CF throughout one's life. For example, stress can hinder attentional shifting in infants (Seehagen, Schneider, Rudolph, Ernst, & Zmyj, 2015) and task-switching abilities in young adults (Plessow, Kiesel, & Kirschbaum, 2012). Moreover, the brain is particularly vulnerable to toxic stress during early life and older adulthood (Lupien, McEwen, Gunnar, & Heim, 2009), so preventative interventions are indicated during these life stages. Research suggests that mindfulness practice and exercise may be efficacious in reducing stress (Vatansever, Wang, & Sahakian, 2021) and boosting EF (Lerche et al., 2018). Notably, exercises incorporating mindfulness, like Tai-Chi and Taekwondo, are more effective in improving EFs compared to standard resistance and aerobic exercises (Diamond & Ling, 2020). One explanation is that mindful exercises demand greater cognitive control, but further research is needed the clarify mechanistic relationships between CF, mindfulness, and general well-being.

### Language factors

The relationship between bi-/multilingualism and EFs, including CF, has generated significant scientific discussion. Studies suggest that bilinguals maintain constant activations for both their languages (Thierry & Wu, 2007) and these continuously active language representations compete for selection during language use, necessitating monitoring and control from bilinguals to achieve successful communication (Valian, 2015). Mechanisms of selection, inhibition and shifting operate in tandem to manage interference but also facilitate language switching when necessary (Gallo, Novitskiy, Myachykov, & Shtyrov, 2021). Clearly, bilinguals need to exercise extensive linguistic control to use their languages effectively, which may be related to domain-general cognitive control, as measured in EF tasks. Despite active research, results remain inconclusive. Several behavioral and neuroimaging studies report better EF performance in bilinguals (Barac, Moreno, & Bialystok, 2016), but others dispute the existence of any advantage (Duñabeitia et al., 2014). This lack of consensus may be due in part to inconsistent measurement of bilingualism as a binary variable rather than as a multifactorial continuum, which obscures deeper differences between monolingual and bilingual groups (Kaushanskaya & Prior, 2015). To advance the field, longitudinal studies and training studies that allow for more careful dissection of causal relationships between multilingualism and mental flexibility are needed. Indicatively, short second-language (L2) learning interventions yield benefits for attention switching in both children (Janus, Lee, Moreno, & Bialystok, 2016) and adults (Bak, Long, Vega-Mendoza, & Sorace, 2016). Explicit training in a language-switching paradigm has shown potential transfer outside the linguistic domain, manifesting in reduced switching (Timmer, Calabria, & Costa, 2019) and mixing costs (Liu et al., 2019) in adult bilinguals. Therefore, learning a new language may potentially be an effective strategy for improving CF throughout the lifespan.

## Future directions and conclusions

The protracted developmental trajectory of CF maturation presents both vulnerability to adverse environmental effects and opportunities for intervention. For instance, during the early years CF interventions might prioritize improving the quality of caregiving through parent-based interventions. During adolescence, peer-to-peer influences within the school environment are of particular importance. Therefore, embedding CF-oriented pedagogy alongside team-based approaches that enhance creativity and inventiveness into school programs and assessments may enhance adolescents' CF and their readiness for the future (Stad, Wiedl, Vogelaar, Bakker, & Resing, 2019).

Maintaining healthy lifestyle habits that boost CF can offer lasting advantages for adults. However, the real challenge often lies in cultivating these habits and sustaining engagement. While gamification techniques might enhance participation in intervention programs (Kappen, Mirza-Babaei, & Nacke, 2020), addressing underlying factors influencing lifestyle choices – like socioeconomic disparities, work-life balance, and social ties – requires broader societal change.

Game-based CF training has potential to enhance CF across the lifespan and may be particularly appealing to children and adolescents (Johann & Karbach, 2020). However, challenges for this field include the age-appropriate adaptation of tasks and ensuring far transfer of training benefits to real-life scenarios. Recent studies have developed innovative paradigms to address these issues, aiming to translate lab-based paradigms to games with superior transfer in real-life contexts, also in clinical trials and practice (Hauser, Iannaccone, Walitza, Brandeis, & Brem, 2015; Langley et al., 2023). In twin studies it has been shown that CF is impacted to a greater extent by environmental factors in contrast to genetic ones, which have been shown to be of relatively low influence, particularly when compared to other EF tasks, for example working memory (Lee et al., 2012). This suggests that CF may be a target which could be improved through training.

In summary, CF skills can be fostered and improved at all life stages, though different interventions may be suitable at each age. To harness this potential, more research is required on CF training to address inconsistent findings and ambiguous transfer effects (Dougherty, Hamovitz, & Tidwell, 2016). For example, one study has shown that CF is separable from EF (Feng et al., 2022), as such training of CF may not have far transfer to other EFs. Nevertheless, many processes such as adaptive learning require CF, and this would be a core component of other EFs, for example problem solving. Therefore, there may be some transfer of CF training to other EFs. Given the importance of CF for lifelong learning, problem-solving, and the mental health of individuals (Buttelmann & Karbach, 2017), further research is essential to better understand associated brain plasticity mechanisms, and to broaden our understanding of the construct and its malleability by social and environmental factors across the lifespan. Furthermore, due to the importance of CF for learning and problem-solving, greater attention needs to be focused on deficits in CF in patients with mental health disorders. It may be possible to improve CF and therefore the impact of impairments in CF may be mitigated if detected early in patients by psychiatrists and psychologists.
